# Safety and applicability of a pre-stage public access ventilator for trained laypersons: a proof of principle study

**DOI:** 10.1186/s12873-017-0150-5

**Published:** 2017-12-04

**Authors:** Patricia Fuchs, Juliane Obermeier, Svend Kamysek, Martin Degner, Hannes Nierath, Henning Jürß, Hartmut Ewald, Jens Schwarz, Martin Becker, Jochen K. Schubert

**Affiliations:** 1 Department of Anaesthesiology and Intensive Care Medicine, Rostock University Medical Centre, Schillingallee 35, 18057 Rostock, Germany; 2 Institute for General Electrical Engineering, University of Rostock, 18059 Rostock, Germany; 3 Sensatronic GmbH Wismar, 23970 Wismar, Germany

**Keywords:** Prototype respirator, Pressure controlled ventilation, Airway occlusion, Lay resuscitation

## Abstract

**Background:**

Contemporary resuscitation guidelines for basic life support recommend an immediate onset of cardiac compressions in case of cardiac arrest followed by rescue breaths. Effective ventilation is often omitted due to fear of doing harm and fear of infectious diseases. In order to improve ventilation a pre-stage of an automatic respirator was developed for use by laypersons.

**Methods:**

Fifty-two healthy volunteers were ventilated by means of a prototype respirator via a full-face mask in a pilot study. The pre-stage public access ventilator (PAV) consisted of a low-cost self-designed turbine, with sensors for differential pressure, flow, FO_2_, FCO_2_ and 3-axis acceleration measurement. Sensor outputs were used to control the respirator and to recognize conditions relevant for efficiency of ventilation and patients’ safety. Different respiratory manoeuvres were applied: a) pressure controlled ventilation (PCV), b) PCV with controlled leakage and c) PCV with simulated airway occlusion. Sensor signals were analysed to detect leakage and airway occlusion. Detection based upon sensor signals was compared with evaluation based on clinical observation and additional parameters such as exhaled CO_2_.

**Results:**

Pressure controlled ventilation could be realized in all volunteers. Leakage was recognized with 93.5% sensitivity and 93.5% specificity. Simulated airway occlusion was detected with 91.8% sensitivity and 91.7% specificity.

**Conclusion:**

The pre-stage PAV was able to detect potential complications relevant for patients’ safety such as leakage and airway occlusion in a proof of principle study. Prospectively, this device provides a respectable basis for the development of an automatic emergency respirator and may help to improve bystander resuscitation.

**Electronic supplementary material:**

The online version of this article (10.1186/s12873-017-0150-5) contains supplementary material, which is available to authorized users.

## Background

Sudden cardiac arrest is one of the major causes of death in Europe [1, 2]. Survival and neurological favourable outcome after cardiac arrest depend on several factors like early recognition, immediate bystander cardio-pulmonary resuscitation (CPR) and high quality CPR including effective chest compressions. Successful resuscitation in case of ventricular fibrillation (VF) cardiac arrest is facilitated by early electrical defibrillation [3, 4].

The importance of oxygenation and ventilation during lay CPR has been discussed controversially over years [5–7]. During respiratory and cardiac arrest ventilation and circulation and thereby oxygen delivery is interrupted. Continued cell metabolism and oxygen consumption leads to decreased oxygen content and critical oxygen supply for vital organs. Oxygen supply for brain and heart is an important variable that determines success probability of cardio-pulmonary resuscitation in cardiac arrest especially of hypoxic origin and during prolonged CPR. Additionally, ventilation is important for carbon dioxide elimination and cardio-pulmonary arrest thus results in respiratory acidosis. Focus of the new ERC guidelines (European Resuscitation Council) for basic life support (BLS) published in 2015 again is the recommendation of an immediate initiation of cardiac compressions by laypersons in case of out of hospital cardiac arrest [4]. Trained CPR providers should perform chest compressions combined with additional rescue breaths. From a pathophysiological point of view, oxygenation and ventilation remain essential to improve outcomes particularly in cardiac arrest of non-cardiac respectively respiratory origin or prolonged resuscitation efforts. Rescue breaths as well as chest compressions are critical for successful resuscitation in these cases [4].

However, 53% of cardiac arrests were in fact witnessed, but only 32% of witnesses performed conventional CPR as reported by Sasson [8]. Possible reasons to withhold resuscitation efforts could be constraints of laypersons to do harm or fear of infectious diseases by performing mouth-to-mouth ventilation [9]. From different studies, it is also known that the effectiveness of ventilation by laypersons is significantly reduced if the training dates back long time ago. Therefore, if willingness to ventilation by laypersons does not exist chest compressions only are recommended [10, 11].

Unlike public access automated external defibrillators (AEDs) or treatment of VF, respirators for effective and safe ventilation are not yet available for use by laypersons during CPR. The concept of public access defibrillators might serve as an exemplary, successful model for life-saving technology applied by laypersons. Early defibrillation can be achieved through CPR providers using AEDs which have already been installed in many public institutions or places. These AEDs can be used safely both by trained laypersons and professional first aid responders [3, 12].

The intention of this project was to develop an automated ventilator in order to support laypersons, who are willing to perform rescue ventilation in patients with sudden cardiac arrest. The functional prototype of the automated public access ventilator (PAV) was conceived as a turbine combined with smart sensor technology. For patients’ safety, reliable detection of malfunction, leakage and airway occlusion is mandatory. Therefore, safety and applicability of the newly developed pre-stage ventilator were evaluated as a first step in healthy volunteers in this proof of principle study.

## Methods

### Study design and participants

This study was designed as a single-centre observational study and carried out in accordance with the ‘Declaration of Helsinki’. After approval of the ethics committee from Rostock University Medical Centre (approval N°: A 2014–0015) and after having obtained written informed consent, 52 healthy volunteers were enrolled into the study. Demographic data from 52 healthy volunteers (28 males, 24 females; aged between 20 and 58 years) are shown in Table 1.

**Table 1:** Demographic characteristics of healthy volunteers

	Total	Men	Women
Number (*n*, %)	52	28 (54)	24 (46)
Age [years] (mean ± SD)	31.9 ± 9.5	32.6 ± 10.0	31.0 ± 8.7
Height [cm] (mean ± SD)	176.1 ± 9.6	181.6 ± 8.0	169.6 ± 7.0
Weight [kg] (mean ± SD)	72.4 ± 14.3	80.0 ± 13.3	63.6 ± 9.5
BMI (mean ± SD)	23.2 ± 3.1	24.2 ± 3.3	22.1 ± 2.4

*SD* standard deviation, *BMI* body mass index

### Prototype setting

**Fig. 1:**
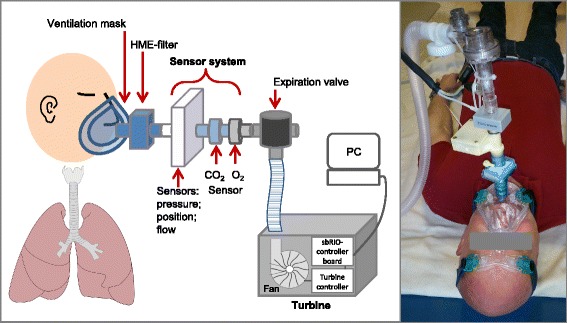
Schematic diagram of sensor prototype and photograph of experimental setup

The pre-stage PAV consisted of a low-cost self-designed turbine and sensors (mainly developed by Sensatronic GmbH) for measuring differential pressure, flow, 3-axis acceleration together with mainstream FO_2_ (fraction of oxygen) and FCO_2_ (fraction of carbon dioxide) with high time resolution (Fig. 1). The turbine was equipped with a brushless motor and works with a fast pressure directed closed loop control to realize respiration profiles without mechanical valves. The maximal turbine flow was 130 l/min at a power consumption of 38 W. The sensors used were based on following measuring principles:

- Flow: anemometric
- Pressure: piezoresistive
- FO_2_: electrochemical
- FCO_2_: non-dispersive infrared absorption (NDIR)
- Position: 3-acis accelerometer (micro electro mechanical system, MEMS).

The combination of different sensors in the pre-stage PAV prototype induced some additional dead space. As PCO_2_ remained constant, this additional dead space was not relevant for ventilation.

A well-directed control of the pre-stage PAV is based on the combination of multiple sensor data and realized by a stand-alone sbRIO-9632 controller board (National Instruments Inc., Munich, Germany). The hardware adaption, signal acquisition and data analysis as well as the complex control of the respirator were carried out by the Institute for General Electrical Engineering of the University of Rostock.

### Experimental protocol

The measurements were performed after the volunteers rested for 15 min, while they received instructions by the tutor. All volunteers were placed in a supine position on a blanket and were ventilated by means of the prototype respirator via a full-face mask with fixation (Ultra Mirage, ResMed GmbH & Co. KG, Germany) connected to the sensor system. Medium and large size masks were used for women and men according to the optimal facial fit. To ensure volunteers’ safety the experimental setting was observed by a physician. Pressure controlled ventilation was performed with inspiratory pressure (p_insp_) 1.5 kPa, positive end expiratory pressure (PEEP) 0 kPa, respiratory rate (RR) 10 breaths/min, I:E = 1:2, and inspiratory oxygen fraction (F_i_O_2_) 0.21.

In order to evaluate the safety and applicability of the pre-stage PAV, three different respiratory manoeuvres were applied for the duration of 1 min, respectively:

- Pressure controlled ventilation (PCV)
- PCV with controlled leakage
- PCV with simulated airway occlusion

During a second study phase these three respiratory manoeuvres were performed under reduced lung compliance induced by wrist weights (14 kg) placed on the chest.

Pressure controlled ventilation was performed after instructing the participants to stop breathing spontaneously and to tolerate ventilation.

For performance of a reproducible leakage manoeuvre, two mask ports (7 mm^2^ each) were opened after 2–3 ventilations, remained open during the following 5 ventilations and were closed again subsequently. The airway occlusion manoeuvre was performed after a resting phase of 5 min. After 2–3 ventilations the participants were asked to close their upper-airway actively for 3 ventilations and to open the airway again afterwards.

### Data acquisition and statistical analysis

During ventilation manoeuvres all raw data as well as the analysed results were logged continuously by the sbRIO-9632 based prototype device. Data analysis and visualization were realized by means of a MatLab-based algorithm established by the Institute for General Electrical Engineering.

Features based on time-resolved integrations of flow and pressure values were used to detect leakage and airway occlusion, respectively. These complications were detected for each breath by the sensor based algorithms of the PAV prototype. Comparisons were done with the evaluation based on clinical observation considering multi-parameter assessment such as adequate generation of flow and pressure as well as exhaled O_2_ and CO_2_ signals.

The criteria for detection of leakage and airway occlusion are given in Table 2.

**Table 2:** Criteria for detection of different manoeuvres

	Mathematical algorithm	Clinical observation
Leakage	Integration (time) of expiratory and inspiratory flow for volume estimation (V_exp_ and V_insp_)	Curve characteristics:
		V_insp_ > V_exp_
		p_eff_ ≠ p_insp_
		Flow_insp_ > 50 l/min
Airway occlusion	Integration (time) of expiratory and inspiratory pressure	Curve characteristics:
		ETCO_2_ ≤ 2%; FO_2_ = constant
		p_eff_ = p_insp_
		Flow ≪ 10 l/min

*V*_*exp*_ expiratory volume, *V*_*insp*_ inspiratory volume, *ETCO*_*2*_ end tidal fraction of carbon dioxide, *FO*_*2*_ oxygen fraction, *p*_*eff*_ effective pressure, *p*_*insp*_ = inspiratory pressure, *Flow*_*insp*_ = inspiratory flow

Statistical analysis was performed with SIGMAPLOT 10 (Systat Software GmbH, Erkrath, Germany). Receiver operating characteristics (ROC) were generated to estimate the sensitivity and specificity of the PAV system in comparison to medical evaluations. The cut-off values were based on feature extraction from temporal integration of expiratory and inspiratory flow (for leakage) as well as from temporal integration of expiratory and inspiratory pressure (for airway occlusion).

## Results

Pressure controlled ventilation was applied in all volunteers according to inspiratory pressure and respiratory rate set to 1.5 kPa and 10 breaths/min, respectively. Resulting tidal volumes were in the range of 8–12 ml/kg.

**Fig. 2:**
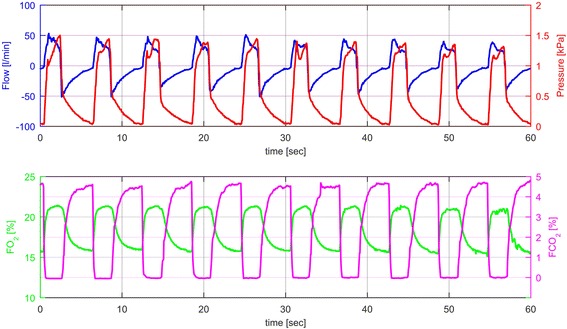
Sensor outputs during PCV without any intervention in one healthy volunteer characterized by typical curves for flow, pressure, FO_2_ and FCO_2_ recorded over one minute (blue line = flow [l/min]; red line = pressure [kPa]; green line = FO_2_ [%]; magenta line = FCO_2_ [%])

**Fig. 3:**
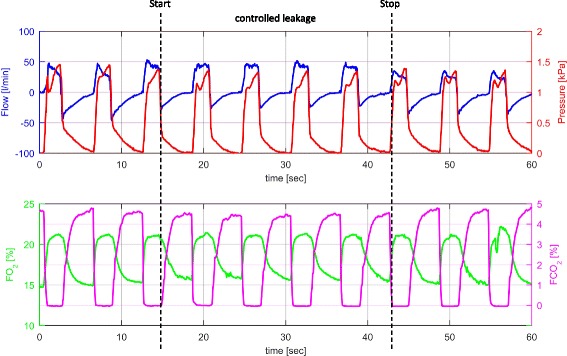
Sensor outputs during PCV and controlled leakage according to the study protocol in one healthy volunteer characterized by curves for flow, pressure, FO_2_ and FCO_2_ recorded over one minute (black dashed lines = start and stop of controlled leakage; blue line = flow [l/min]; red line = pressure [kPa]; green line = FO_2_ [%]; magenta line = FCO_2_ [%])

**Fig. 4:**
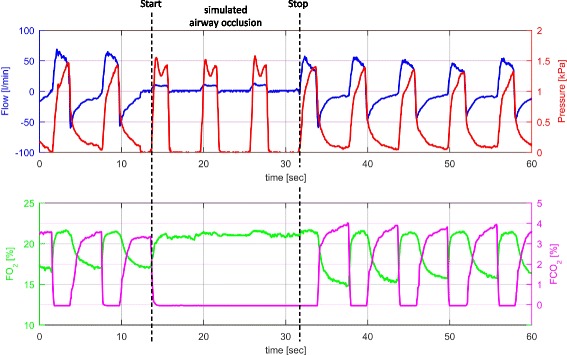
Sensor outputs during PCV and simulated airway occlusion according to the study protocol in one healthy volunteer characterized by curves for flow, pressure, FO_2_ and FCO_2_ recorded over one minute (black dashed lines = start and stop of simulated airway occlusion; blue line = flow [l/min]; red line = pressure [kPa]; green line = FO_2_ [%]; magenta line = FCO_2_ [%])

Figures 2, 3 and 4 show results obtained from different sensor outputs during the applied respiratory manoeuvres. The graphs illustrate flow and pressure as well as FO_2_ and FCO_2_ values over time.

Figure 2 describes pressure controlled ventilation without any intervention in one healthy volunteer characterized by typical curves for flow, pressure, FO_2_ and FCO_2_ recorded over one minute.

Figure 3 illustrates PCV with controlled leakage according to the study protocol. As a consequence during leakage expiratory flow decreased, while the FO_2_ and FCO_2_ curves did not change markedly. During the leakage manoeuvre expiratory volumes decreased compared to inspiratory volumes. Compared to ventilation without leakage the decrease of expiratory tidal volume was 25 to 35%.

Figure 4 shows PCV interrupted by an airway occlusion manoeuver according to the study protocol. During upper airway occlusion, no typical in- and expiratory flow curve was generated and therefore FO_2_ and FCO_2_ remained on a constant level. The pre-set airway pressure (p_insp_) was always reached, but never exceeded 1.5 kPa. With respect to the criteria for complete airway occlusion being defined in Table 2 (e.g. ETCO_2_ ≤ 2%), 80% of all volunteers were able to close their airways entirely.

All results from the second study phase with PCV manoeuvers under reduced lung compliance are presented in Additional file 1: Figure S1; Additional file 2: Figure S2; and Additional file 3: Figure S3.

**Fig. 5:**
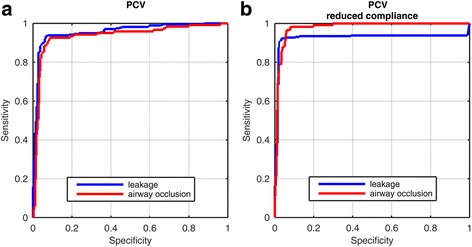
Receiver operating characteristic (ROC) curves concerning recognition of the two interventions during PCV: controlled leakage (blue line) and simulated airway occlusion (red line) with unrestricted lung compliance (**a**) and with reduced lung compliance (**b**) induced by weights (14 kg) placed on the chest

Leakage and airway occlusion were recognized with over 92% sensitivity and specificity. Fig. 5 presents the corresponding ROC curves. Table 3 summarizes relevant values for sensitivity and specificity of the ROC curves for different manoeuvres.

**Table 3:** Results from receiver operating characteristics

Manoeuvers	Sensitivity [%]	Specificity [%]
PCV with controlled leakage	93.5	93.5
PCV with simulated airway occlusion	91.8	91.7
PCV with leakage, reduced lung compliance	92.4	96.6
PCV with airway occlusion, reduced lung compliance	96.6	94.0

*PCV* pressure controlled ventilation

## Discussion

In this pilot study, we demonstrated safety and applicability of an innovative pre-stage public access ventilator. The compact PAV system consisting of a turbine, sensors (flow, pressure, FO_2_, FCO_2_, 3-axis accelerometer) and a control unit was able to provide pressure controlled ventilation in healthy volunteers. Detection of potential complications relevant for patient’s safety such as leakage and airway occlusion was realized with sensitivity and specificity > 92%.

Although only half of cardiac arrests were witnessed, layperson CPR has been shown to be an important predictor of increased survival to hospital discharge [13, 14]. However, laypersons are often in doubt if they can perform effective ventilation. Aggravating this situation, there is a risk for gastric air insufflation resulting in a potential risk for pulmonary aspiration [15, 16]. In the current literature, there is only a small number of publications addressing the effectivity of ventilation by laypersons [17–19]. In 2003, when the ERC guidelines recommended explicitly rescue breaths for CPR, Woollard et al. used a manikin model of cardiac arrest to compare skill performance in untrained laypersons receiving compressions-only or standard telephone CPR [17]. A number of subjects did not open the airway and more than 75% in the standard telephone group failed to deliver two effective initial rescue breaths. Only 17% provided an adequate inflation volume for subsequent breaths. The problem of delays to first compression and poor performance of airway opening and ventilation skills was also reported by Kellermann in a simulated cardiac arrest scenario [18]. The use of a public access respirator could support trained laypersons by effective automated non-invasive ventilation during CPR.

Recently, Nitzschke et al. tested the effect of a CPR assist device with an AED synchronised with a commercially available ventilator on the CPR performance of emergency medical staff [19]. They recommended their study device for ALS providers with limited experience only. Instead of using an expensive ventilator for professional medical staff, our intention was to develop a cost effective innovative functional prototype of a PAV to realize a wide distribution in public places and institutions in order to support laypersons. Moreover, an advantage of the turbine used in our study in contrast to the commercially available ventilators is its independency of any kind of additional gas supply.

To assure patient’s safety and to avoid possible harm, e.g. gastric insufflation, the PAV-system has to be able to recognize and properly manage potential complications such as leakage or the status when airways are closed.

As PetCO_2_ may be low under resuscitation conditions [20, 21], in this study we used a mathematical algorithm for the detection of airway occlusion which was based on P_insp_ and did not include PetCO_2_ as a crucial parameter. Compared to medical observation that was based on PetCO_2_, FO_2_, P_insp_ and flow, the sensor system was able to detect leakage and airway occlusion with high sensitivity and specificity over 92%. As a fundamental requirement for support systems used by trained laypersons the sensor prototype could detect whether the airways were closed. This is a necessary prerequisite for immediate interruption of ventilation in those cases. In addition, head position could be monitored by the PAV by means of the 3-axis accelerometer. Thereby, users could be told to perform a head tilt/chin lift manoeuver to open the airways.

In this setting we tried to simulate elevated intrathoracic pressures and reduced lung compliance which may occur when chest compressions are performed during resuscitation [22–24]. As we used pressure controlled ventilation with constant p_insp_ and constant respiratory rate, significantly reduced lung compliance could have led to decreased tidal volumes and minute ventilation volumes. In contrast to what should have been expected, tidal volumes remained constant when weight was placed onto volunteers’ chests and sensitivity and specificity for recognition of airway closure and leakage was even better in this setting. Possible explanations for these unexpected findings could be that primarily thoracic and not lung compliance was reduced when weight was put onto volunteers’ chests [25]. In addition, participants may involuntarily have compensated the reduced thoracic compliance through increased (spontaneous) breathing efforts.

Generally, both volume and pressure controlled ventilation can be used in emergency respirators. In case of pressure controlled ventilation a strict monitoring of ventilation pressure is mandatory to avoid gastric air insufflation. As airway pressure > 2.0 kPa causes opening of the oesophageal sphincter, we limited the maximum inspiratory pressure to 1.5 kPa in this study.

Another crucial problem in mask ventilation is leakage occurring when a tight fit of the mask cannot be achieved for any reason. As the PAV is to be used by trained laypersons it is highly probable that leakage will occur [4]. Leakage in ventilation is defined as the difference between inspiratory and expiratory tidal volumes. The PAV, therefore, analysed the difference between inspiratory and expiratory flows. This data was then used to determine the percentage of leakage with respect to inspired tidal volume. For non-invasive mask ventilation, commercially available ventilation systems can compensate more than 100% of leakage. In our study, we used a small, standardized and reproducible leakage of 25–35% regarding inspired tidal volume. Although this leakage could easily be compensated through the turbine and it did not significantly impair ventilation, the pre-stage PAV reliably recognized the expiratory decrease of tidal volume. For use in emergency settings, leakage recognition could be used together with 3-axis accelerometer output to guide users to readjust mask fitting and head position.

An important limitation of this study was the fact that the pre-stage PAV could not be tested in a real resuscitation situation. The study setup in volunteers did not allow a combination of ventilation and chest compressions. Furthermore, healthy volunteers cannot be regarded as patients with respect to unimpaired spontaneous breathing, protective functions as well as lung compliance. Even a pig model, which is often used as nearly equivalent to human organism had to be excluded due to the different anatomy concerning the epiglottis [26].

Prospectively, sensor data (flow, pressure, FO_2_, FCO_2_, 3-axis accelerometer) could be used for an adapted feedback algorithm [27] integrated in a PAV to guide laypersons in an audio-visually controlled way comparable to well-established automatic external defibrillators (AED) with voice instructions [3, 12].

A potential area for future investigation could be optimization and miniaturization of the sensor system and adaption of the algorithm. In the next step, cardiopulmonary resuscitation studies in life support training manikins supported by optimized PAV are necessary, followed by field studies in patients. In this context, an appropriate teaching and training of BLS providers has to be established sustainably [28].

## Conclusion

In conclusion, the pre-stage PAV provides a respectable basis for the development of an automatic emergency respirator. Evaluation of ventilation parameters such as tidal volume, breathing frequency and inspiratory pressure indicated effective ventilation by the examined device. Safety aspects like leakage and airway occlusion were detected effectively.

## Additional files

Additional file 1: Figure S1. Sensor outputs during PCV manoeuvre under reduced lung compliance. Typical curves for flow, pressure, FO_2_ and FCO_2_ are presented over one minute from one healthy volunteer (blue line = flow [l/min]; red line = pressure [kPa]; green line = FO_2_ [%]; magenta line = FCO_2_ [%]). (PDF 1133 kb)

Additional file 2: Figure S2. Sensor outputs during PCV under reduced lung compliance and controlled leakage. According to the study protocol curves for flow, pressure, FO_2_ and FCO_2_ were recorded over one minute from one healthy volunteer (black dashed lines = start and stop of controlled leakage; blue line = flow [l/min]; red line = pressure [kPa]; green line = FO_2_ [%]; magenta line = FCO_2_ [%]). (PDF 1127 kb)

Additional file 3: Figure S3. Sensor outputs during PCV under reduced lung compliance and simulated airway occlusion. Sensor curves for flow, pressure, FO_2_ and FCO_2_ are presented over one minute from one healthy volunteer (black dashed lines = start and stop of simulated airway occlusion; blue line = flow [l/min]; red line = pressure [kPa]; green line = FO_2_ [%]; magenta line = FCO_2_ [%]). (PDF 1082 kb)

## Abbreviations

AED: Automated external defibrillator
BLS: Basic life support
BMI: Body mass index
CPR: Cardio-pulmonary resuscitation
ERC: European Resuscitation Council
ETCO_2_: End tidal fraction of carbon dioxide
FCO_2_: Carbon dioxide fraction
F_i_O_2_: Inspiratory oxygen fraction
Flow_insp_: Inspiratory flow
FO_2_: Oxygen fraction
I:E: Ratio of inspiration to expiration
MEMS: Micro electro mechanical system
NDIR: Non-dispersive infrared absorption
PAV: Public access ventilator
PCV: Pressure controlled ventilation
PEEP: Positive end expiratory pressure
p_eff_: Effective pressure
p_insp_: Inspiratory pressure
ROC: Receiver operating characteristics
RR: Respiratory rate
SD: Standard deviation
V_exp_: Expiratory volume
VF: Ventricular fibrillation
V_insp_: Inspiratory volume
