# Supplementary figures and images for: Safety and applicability of a pre-stage public access ventilator for trained laypersons: a proof of principle study
Source: BMC Emerg Med. 2017 Dec 4;17:37. doi: 10.1186/s12873-017-0150-5 (PMC5716260; doi:10.1186/s12873-017-0150-5)

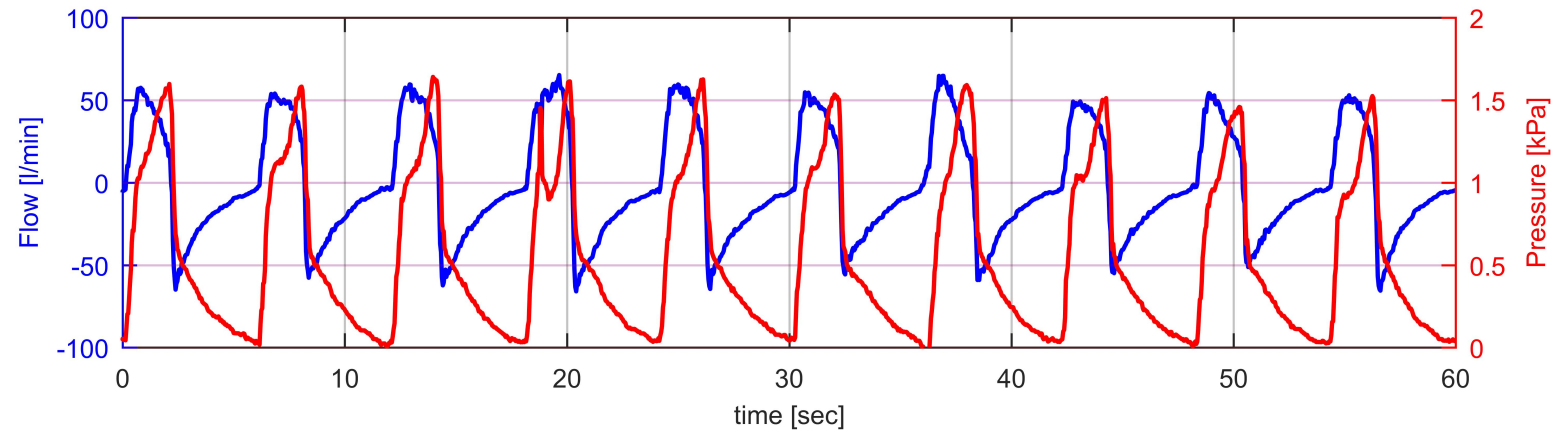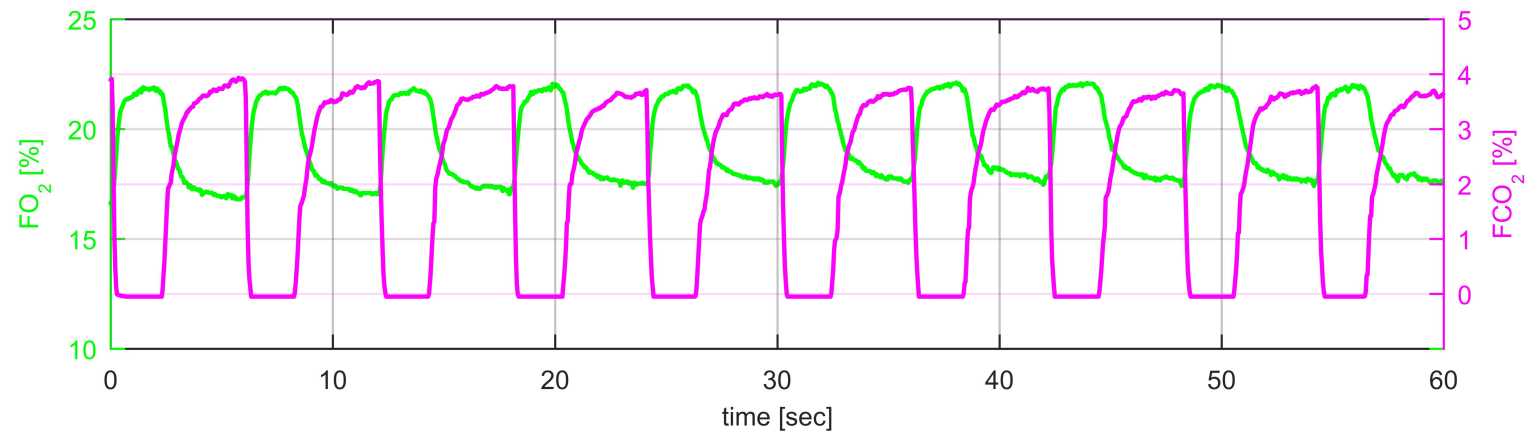

Supplement: Supplementary file 1 — Sensor outputs during PCV manoeuvre under reduced lung compliance. Typical curves for flow, pressure, FO2 and FCO2 are presented over one minute from one healthy volunteer (blue line = flow [l/min]; red line = pressure [kPa]; green line = FO2 [%]; magenta line = FCO2 [%]). (PDF 1133 kb) [file 12873_2017_150_MOESM1_ESM.pdf]

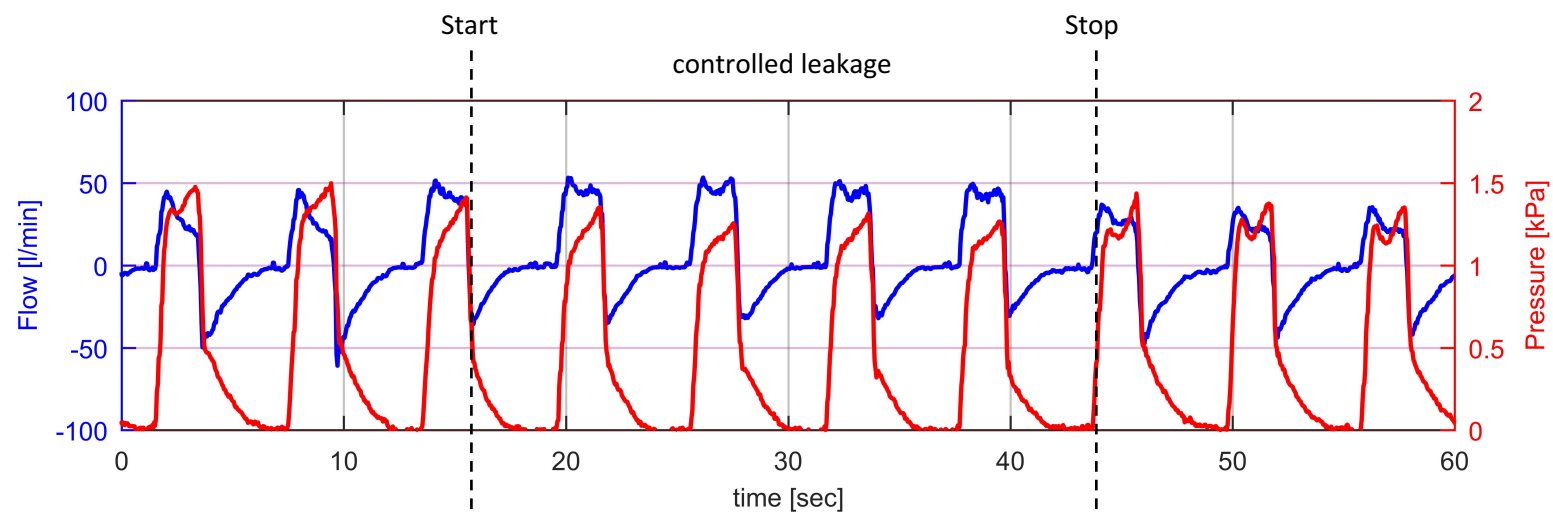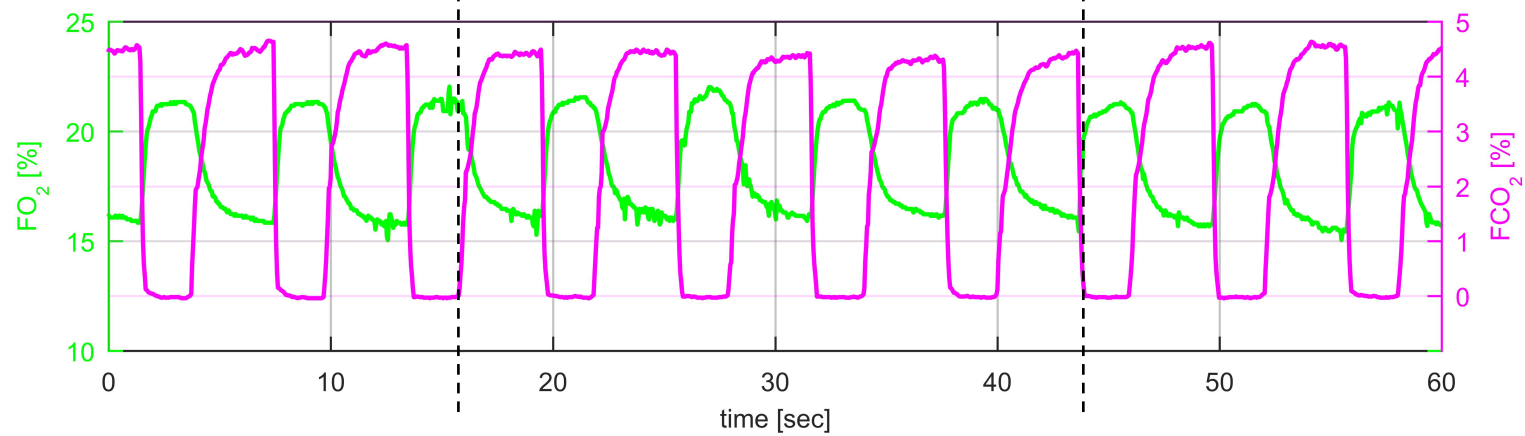

Supplement: Supplementary file 2 — Sensor outputs during PCV under reduced lung compliance and controlled leakage. According to the study protocol curves for flow, pressure, FO2 and FCO2 were recorded over one minute from one healthy volunteer (black dashed lines = start and stop of controlled leakage; blue line = flow [l/min]; red line = pressure [kPa]; green line = FO2 [%]; magenta line = FCO2 [%]). (PDF 1127 kb) [file 12873_2017_150_MOESM2_ESM.pdf]

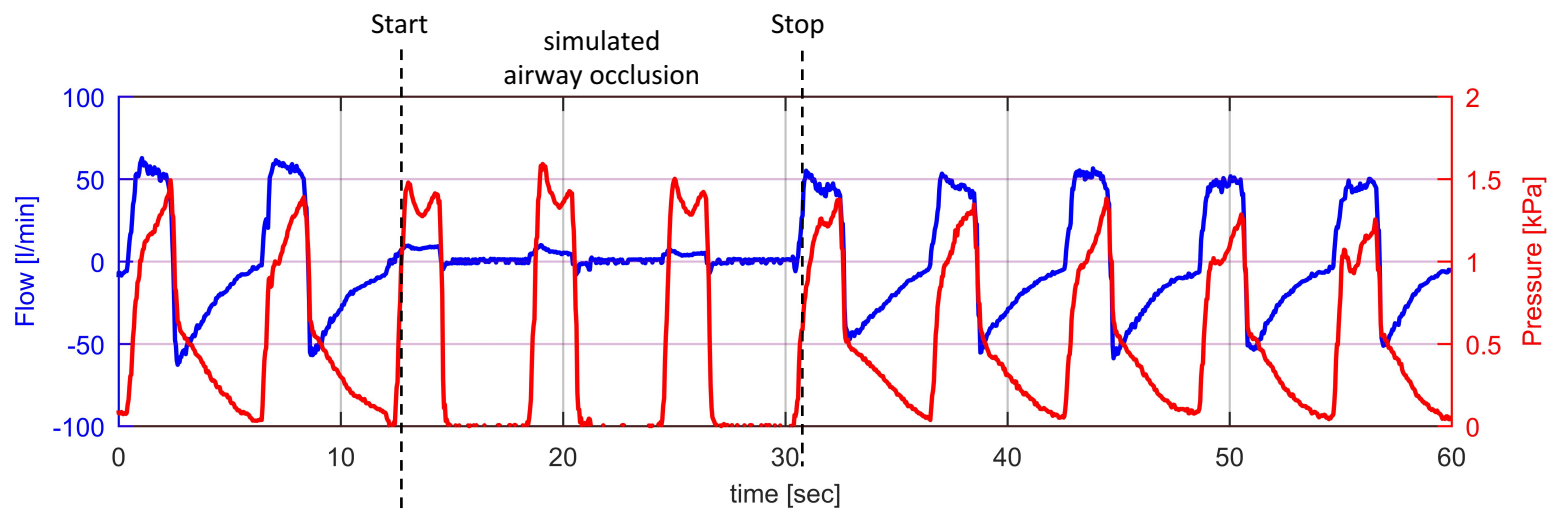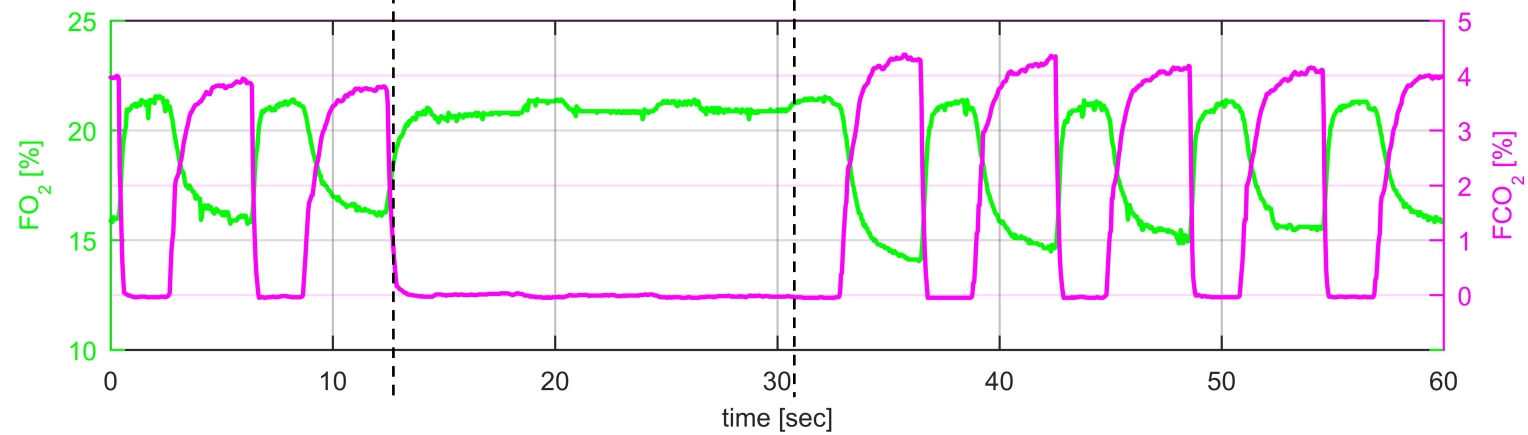

Supplement: Supplementary file 3 — Sensor outputs during PCV under reduced lung compliance and simulated airway occlusion. Sensor curves for flow, pressure, FO2 and FCO2 are presented over one minute from one healthy volunteer (black dashed lines = start and stop of simulated airway occlusion; blue line = flow [l/min]; red line = pressure [kPa]; green line = FO2 [%]; magenta line = FCO2 [%]). (PDF 1082 kb) [file 12873_2017_150_MOESM3_ESM.pdf]
